# Triglyceride glucose index is associated with cerebral small vessel disease burden and cognitive impairment in elderly patients with type 2 diabetes mellitus

**DOI:** 10.3389/fendo.2022.970122

**Published:** 2022-08-05

**Authors:** Zhenjie Teng, Jing Feng, Yanhong Dong, Jing Xu, Xin Jiang, Huifang Chen, Qianqian Qi, Rui Li, Weihong Chen, Peiyuan Lv

**Affiliations:** ^1^ Department of Neurology, Hebei Medical University, Shijiazhuang, China; ^2^ Department of Neurology, Hebei General Hospital, Shijiazhuang, China; ^3^ Department of Endocrinology, Hebei General Hospital, Shijiazhuang, China

**Keywords:** triglyceride glucose index, cerebral small vessel disease, cognitive impairment, insulin resistance, diabetes

## Abstract

**Objective:**

To investigate the relations of Triglyceride glucose (TyG) index with cerebral small vessel disease (CSVD) burden and cognitive function in aged patients with type 2 diabetes mellitus (T2DM).

**Methods:**

A total of 308 elderly patients with T2DM were included in this retrospective study. The standardized Chinese version of Mini-Mental State Examination was used to assess cognitive function. The total CSVD burden score was assessed by combining four imaging markers of CSVD, including the presence of white matter hyperintensity, cerebral microbleeds in the deep, lacunes and enlarged perivascular spaces in the basal ganglia. The TyG index was calculated as the formula of ln [fasting triglyceride (mg/dl) × fasting plasma glucose (mg/dl)/2]. We used logistic regression analysis and mediation analysis to investigate the relations of TyG index with CSVD and cognitive function.

**Results:**

Multivariate binary logistic regression analysis showed that increased TyG index (OR: 2.241; 95% Confidence Interval(CI): 1.439 to 3.490; *P <*0.001), or severe CSVD burden (OR: 2.198; 95% CI: 1.283 to 3.763; *P* = 0.004) was associated with an increased risk of cognitive impairment in elderly patients with T2DM after adjusting for potential confounders. In addition, TyG index was an independent risk factor of severe CSVD burden (OR: 1.472; 95% CI: 1.003 to 2.160; *P* = 0.048) after controlling for potential confounders. Compared with the lowest TyG index tertile, the multivariable-adjusted OR of the highest tertile was 3.298 (95% CI: 1.685 to 6.452; *P* for trend <0.001) for cognitive impairment, 1.933 (95% CI: 1.010 to 3.698; *P* for trend = 0.047) for severe CSVD burden. Mediation analysis found a significant moderating effect of the severe CSVD burden on the association between higher TyG index levels and cognitive impairment.

**Conclusions:**

The increased TyG index is an independent risk factor for cognitive impairment and severe CSVD burden in clinical practice. A proportion of the effect of increased TyG index on cognitive impairment may be due to the aggravation of CSVD burden.

## Introduction

Type 2 diabetes mellitus (T2DM) is a prevalent disease in the aging population and puts a tremendous burden on the global healthcare system (1). Cognitive impairment is increasingly recognized as an important complication of T2DM (2). T2DM is associated with a 1.5-2.8 times increased risk of dementia (3, 4), and approximately 20 percent of patients older than 60 years might have dementia in the T2DM population (2). In particular, T2DM can accelerate the progression from mild cognitive impairment to dementia (2, 5). Moreover, cognitive impairment may result in poor self-management of T2DM, which in turn lead to worse cognitive ability and more cerebrovascular events (6).

The pathogenesis of T2DM-related cognitive impairment is multifactorial, complicated and incompletely understood. One important mechanism might be microvascular dysfunction, which has been proven to cause many organs dysfunction including the brain (7). Cerebral small vessel disease (CSVD) might be visible imaging markers of cerebral microvascular dysfunction (7). Magnetic resonance imaging (MRI) features of CSVD include white matter hyperintensity (WMH), lacunes, cerebral microbleeds (CMBs), enlarged perivascular spaces (EPVS), total brain atrophy and microinfarcts (8). The CSVD burden score, which combines individual imaging markers into a score, may better reflect the severity of CSVD (9).

Insulin resistance has been widely acknowledged to be a core feature of T2DM and shown to be a significant pathogenic mechanism of cognitive impairment (10, 11). Possible pathways proposed to explain the mechanism include endothelial dysfunction, blood-brain barrier (BBB) dysfunction, oxidative stress or inflammation due to failure of cells to respond to insulin in the brain (10). Interestingly, these pathways play a crucial role in the pathogenesis of CSVD (12–14). Triglyceride glucose (TyG) index, a convenient and accessible measure, is considered a surrogate marker for insulin resistance in patients with T2DM (15). However, few studies have explored the association of TyG index with CSVD burden and cognitive function.

Given the importance of insulin resistance and microvascular dysfunction in T2DM-related cognitive impairment. In this research, we attempted to explore whether TyG index is associated with an increased risk of severe CSVD burden or cognitive impairment and whether CSVD burden mediates the effect of TyG index on cognitive function in elderly patients with T2DM.

## Materials and methods

### Participants

A total of 308 eligible patients with T2DM who were admitted to Hebei General Hospital between October 2016 and March 2021 were enrolled in this retrospective study. Inclusion criteria (1): aged 60 years and older; (2) met diagnostic criteria for diabetes according to World Health Organization (16); (3) completed MRI sequences with the 3.0 tesla MR scanner and neuroimaging sequences included T1- and T2-weighted imaging, T2 fluid-attenuated inversion recovery (T2 FLAIR) and susceptibility weighted imaging (SWI); (4) completed cognitive function assessment. Exclusion criteria: (1) subjects with type 1 diabetes mellitus; (2) subjects with acute stroke, myocardial infarction or severe neurologic deficit; (3) subjects with other specific conditions which may be associated with cognitive impairment, such as brain injuries, epilepsy, malignancy, anxiety, depression, hypothyroidism, hyperthyroidism, carbon monoxide poisoning, schizophrenia, anxiety or depression. This study followed the principles in the Declaration of Helsinki and was approved by the Ethical Committees of Hebei General Hospital (NO.2022090).

### Demographic, clinical, and laboratory data collection

The demographic and clinical data included age, gender, years of education, height, weight, systolic blood pressure (SBP), diastolic blood pressure (DBP), previous medical diagnosis, cigarette smoking and alcohol drinking status, treatment with antiplatelet drugs or statin and diabetes medications were collected from in-hospital records stored in electronic databases or by interviewing the patients. Body mass index was calculated as weight (kg) divided by square of height (m^2^). Laboratory examinations, such as fasting plasma glucose (FPG), triglyceride (TG), total cholesterol (TC), high density lipoprotein (HDL), low density lipoprotein (LDL), hemoglobin A1c (HbA1c), uric acid and serum total homocysteine, were obtained after 8 h of overnight fasting and included. The TyG index was calculated as the formula of ln [fasting TG (mg/dl) × FPG (mg/dl)/2] (17).

### Evaluation of CSVD burden score

All patients underwent brain MRI examination using 3.0 tesla magnetic resonance scanners (Signa, GE Healthcare, American). The total CSVD burden score was assessed by combining four imaging markers of CSVD, including the presence of WMH, CMBs in the deep, lacunes and EPVS in the basal ganglia (18). Two trained readers who were blinded to clinical details evaluated these imaging markers based on the STRIVE criteria (8). When there is a dispute on any imaging markers of CSVD, a third reader evaluated the images in order to reach an agreement. The severity of WMH was evaluated according to Fazekas rating scale on T2-weighted imaging and T2 FLAIR images (19). A point was awarded on the CSVD burden score when the Fazekas score of periventricular WMH was 3 or deep WMH was 2 or 3. CMBs in deep (basal ganglia, thalamus, external capsule, internal capsule, corpus callosum, and deep and periventricular white matter) were recorded on SWI using the Microbleed Anatomical Rating Scale (20). A point was awarded on the CSVD burden score in case of the presence of any deep CMBs. Lacunes were defined as small (3-15 mm), round or ovoid, fluid-filled lesions (signal similar to cerebrospinal fluid) on T1- or T2-weighted imaging (8). A point was awarded on the CSVD burden score when one or more lacunes were present. EPVS in basal ganglia were defined as small (<3 mm), linear or round lesions with cerebrospinal fluid-like signals on T2-weighted imaging (8). EPVS in basal ganglia were rated on a semi-quantitative scale and scored as 0 (none), 1 (1-10 EPVS), 2 (11-20 EPVS), 3 (21-40 EPVS), and 4 (>40 EPVS) on one side of the brain (21). A point was awarded on the CSVD burden score when the EPVS score in the basal ganglia was 2 or 3 or 4. We defined severe CSVD burden when the score was greater than 2 (22).

### Evaluation of cognitive function

The standardized Chinese version of Mini-Mental State Examination (MMSE) was used to assess cognitive function for all patients in our study. The levels of education are highly recommended to consider when explaining the results of MMSE (23). Therefore, the cut-off scores for diagnosing cognitive impairment were limited to 17 for illiterate, 20 for patients with 1-6 years of education, and 24 for patients with 7 or more years of education based on the previously published protocol (24).

### Statistical analysis

Continuous variables were expressed as mean (standard deviation) or median (interquartile range) and for categorical variables, as frequency. We used Mann-Whitney U tests, T tests or χ^2^ tests for different variables, as appropriate. Variables with *P* < 0.1 in the above tests were introduced into the multivariate binary logistic regression model to identify independent risk factors for cognitive impairment or severe CSVD burden in elderly patients with T2DM. Tests for trend were conducted with using the tertiles of TyG index levels as a continuous variable by assigning the median values of the tertiles to the variable. The receiver operating characteristic (ROC) curve of TyG index levels was drawn and the optimal cut-off point of TyG index levels in patients with cognitive impairment was predicted according to the maximum value of the Youden Index. SPSS 26.0 (IBM corporation, Armonk, NY) was used for above analyses. *P <*0.05 was considered to be statistically significant.

R, version 4.1.0 (R Foundation for Statistical Computing, Vienna, Austria) was used to perform mediation analysis. The bruceR and mediation packages were used to assess whether severe CSVD burden mediate the relation between higher TyG index levels and cognitive impairment in elderly patients with T2DM. The function can automatically judge the model type and conduct mean centering before model building. The number of bootstrap samples in each analysis was set to 5,000 to obtain a more robust estimate of the effect.

## Results

### Participants characteristics

This retrospective study included 308 eligible aged patients with T2DM. The mean age of patients was 70.6 ± 6.1 years and 48.7% (n = 150) were male. Based on the scores of MMSE, all patients were divided into two groups: no cognitive impairment group (n = 158) and cognitive impairment group (n = 150). The characteristics of all patients between the two groups are presented in **Table 1**. Patients in the cognitive impairment group were significantly older, lower educated and had lower frequencies of metformin usage and higher frequencies of history of stroke or insulin usage than those in no cognitive impairment group (*P <*0.05). The cognitive impairment group presented higher HbA1c, serum tHcy, TyG index and total CSVD burden score, but lower HDL-C than no cognitive impairment group (*P <*0.05).

**Table 1:** Characteristics of the study participants between no cognitive impairment and cognitive impairment group.

Variable	Total(n = 308)	No cognitive impairment group (n=158)	Cognitive impairment group (n=150)	*P* value
Age, median (IQR)), year	71 (66-75)	70 (65-73)	72 (66-76)	0.001*
Sex (male), n (%)	150 (48.7)	73 (46.2)	77 (51.3)	0.368
Education, median (IQR), year	9 (6-12)	9 (6-12)	9 (6-12)	0.038*
Body mass index, median (IQR), kg/m^2^	25.00 (23.05-27.33)	25.40 (23.33-27.43)	24.82 (22.90-27.15)	0.196
Current smoking, n (%)	42 (13.6)	17 (10.8)	25 (16.7)	0.131
Alcohol use, n (%)	31 (10.1)	12 (7.6)	19 (12.7)	0.139
Hypertension, n (%)	232 (75.3)	116 (73.4)	116 (77.3)	0.426
SBP, median (IQR), mmHg	140 (130-153)	138 (129-150)	141 (130-157)	0.077
DBP, median (IQR), mmHg	81 (75-89)	81 (74-88)	81 (75-90)	0.300
Duration of T2DM, median (IQR), year	10 (5-17)	10 (5-14)	10 (4-20)	0.560
HbA1c, median (IQR), %	7.2 (6.5-8.2)	7.0 (6.5-7.8)	7.5 (6.8-8.8)	< 0.001*
Coronary heart disease, n (%)	83 (26.9)	42 (26.6)	41 (27.3)	0.882
History of stroke	132 (42.9)	52 (32.9)	80 (53.3)	< 0.001*
TC, median (IQR), mmol/L	4.28 (3.56-5.09)	4.25 (3.60-4.96)	4.37 (3.49-5.18)	0.771
HDL, median (IQR), mmol/L	1.05 (0.90-1.28)	1.11 (0.95-1.31)	1.01 (0.88-1.21)	0.004
LDL, median (IQR), mmol/L	2.73 (2.17-3.42)	2.72 (2.19-3.38)	2.76 (2.11-3.46)	0.642
Uric acid, median (IQR), umol/L	290.6 (233.5-349.3)	274.4 (233.4-342.6)	297.8 (233.9-354.3)	0.339
Serum tHcy, median (IQR), umol/L	13.6 (11.0-16.5)	12.9 (10.4-15.3)	14.5 (11.7-17.8)	0.001*
Medication				
Antiplatelet, n (%) Statin, n (%) Insulin, n (%) Metformin, n (%) DPP-4 inhibitors, n (%) GLP-1 receptor agonist, n (%)	101 (32.8) 143 (46.4) 113 (36.7) 235 (76.3) 78 (25.3) 56 (18.2)	47 (29.7) 78 (49.4) 49 (31.0) 129 (82.6) 34 (21.5) 30 (19.0)	54 (36.0) 65 (43.3) 64 (42.7) 106 (70.7) 44 (29.3) 26 (17.3)	0.243 0.289 0.034* 0.024* 0.115 0.707
TyG index, mean (SD)	8.99 ± 0.68	8.79 ± 0.57	9.20 ± 0.72	< 0.001*
Total CSVD burden score				
0, n (%) 1, n (%) 2, n (%) 3, n (%) 4, n (%)		31 (19.6) 36 (22.8) 35 (22.2) 34 (21.5) 22 (13.9)	6 (4.0) 14 (9.3) 33 (22.0) 54 (36.0) 43 (28.7)	< 0.001*

*P <0.05. SD, standard deviation; IQR, interquartile range; SBP, systolic blood pressure; DBP, diastolic blood pressure; T2DM, type 2 diabetes mellitus; TC, total cholesterol; HDL, high density lipoprotein; LDL, low density lipoprotein; tHcy, total homocysteine; DPP-4, Dipeptidyl peptidase-4; GLP-1, glucagon-like peptide-1; TyG, triglyceride glucose; CSVD, cerebral small vessel disease.

### Triglyceride glucose index and cognitive impairment

In unadjusted binary logistic regression analysis, increased TyG index was associated with cognitive impairment (OR: 2.811; 95% CI: 1.899 to 4.160; *P <*0.001). In multivariate binary logistic regression analysis, the association remained significant (OR: 2.241; 95% CI: 1.439 to 3.490; *P <*0.001), after adjusting for age, education, SBP, history of stroke, HbA1c, HDL-C, serum tHcy, insulin or metformin usage and severe CSVD burden (**Table 2**).

**Table 2:** The logistic regression analyses between possible predictors and cognitive impairment.

	Univariable analysis		Multivariable analysis^1^	
	OR (95% CI)	*P* Value	OR (95% CI)	*P* Value
Age	1.074 (1.034-1.116)	< 0.001	1.067 (1.021-1.115)	0.004
Education	0.944 (0.894-0.998)	0.041	0.927 (0.871-0.988)	0.019
SBP	1.013 (1.000-1.026)	0.046	1.008 (0.993-1.023)	0.304
HbA1c	1.417 (1.188-1.690)	< 0.001	1.258 (1.028-1.540)	0.026
History of stroke	2.330 (1.468-3.696)	< 0.001	1.683 (0.977-2.899)	0.061
HDL	0.307 (0.125-0.749)	0.010	0.421 (0.143-1.235)	0.115
Serum tHcy	1.243 (0.850-1.817)	0.024	1.023 (0.994-1.052)	0.127
Severe CSVD burden	3.334 (2.089-5.319)	< 0.001	2.198 (1.283-3.763)	0.004
Medication				
Insulin Metformin	1.655 (1.037-2.641) 0.542 (0.317-0.924)	0.034 0.025	1.458 (0.840-2.531) 0.630 (0.337-1.176)	0.180 0.147
TyG index	2.811 (1.899-4.160)	< 0.001	2.241 (1.439-3.490)	< 0.001

SBP, systolic blood pressure; HDL, high density lipoprotein; tHcy, total homocysteine; CSVD, cerebral small vessel disease; TyG, triglyceride glucose.

^1^Adjusted with P < 0.10 in the **Table 1** (age, education, SBP, HbA1c, history of stroke, HDL, serum tHcy, severe CSVD burden, insulin or metformin usage, and TyG index).

Higher TyG index was associated with a significantly higher risk of cognitive impairment in elderly patients with T2DM when adjusting for age, sex and education (OR: 4.709; 95% CI: 2.515 to 8.817; *P* for trend <0.001) and after further adjustment for hypertension, history of stroke, SBP, HbA1c, HDL-C, insulin or metformin usage and serum tHcy (OR: 3.298; 95% CI: 1.685 to 6.452; *P* for trend <0.001) in the highest tertile of TyG index levels compared with the lowest (**Table 4**).

### Triglyceride glucose index and CSVD burden

In unadjusted binary logistic regression analysis, elevated TyG index was associated with severe CSVD burden (OR:1.582; 95% CI: 1.123 to 2.227; *P* = 0.009). In multivariate binary logistic regression analysis, TyG index was an independent risk factor of severe CSVD burden (OR: 1.472; 95% CI: 1.003 to 2.160; *P* = 0.048), after further adjustment of potential confounders, such as age, sex, hypertension, SBP, history of stroke, metformin usage and HDL-C (**Table 3**).

**Table 3:** The logistic regression analyses between possible predictors and severe CSVD burden.

	Univariable analysis		Multivariable analysis^1^	
	OR (95% CI)	*P* Value	OR (95% CI)	*P* Value
Age	1.051 (1.012-1.091)	0.010	1.040 (1.000-1.082)	0.052
Sex (male)	1.642 (1.046-2.575)	0.031	1.509 (0.884-2.576)	0.131
Education	0.970 (0.919-1.024)	0.270	—	—
Body mass index	1.038 (0.970-1.111)	0.280	—	—
Current smoking	1.347 (0.695-2.610)	0.378	—	—
Alcohol use	1.690 (0.790-3.614)	0.176	—	—
Hypertension	2.157 (1.262-3.687)	0.005	1.939 (1.060-3.548)	0.032
SBP	1.016 (1.003-1.029)	0.014	1.010 (0.996-1.024)	0.164
DBP	1.014 (0.994-1.034)	0.160	—	—
Duration of T2DM	0.994 (0.968-1.021)	0.656	—	—
HbA1c	1.082 (0.924-1.028)	0.329	—	—
Coronary heart disease	0.836 (0.505-1.382)	0.485	—	—
History of stroke	2.872 (1.799-4.586)	< 0.001	2.234 (1.357-3.677)	0.002
TC	0.961 (0.790-1.686)	0.689	—	—
HDL	0.291 (0.119-0.711)	0.007	0.514 (0.185-1.428)	0.202
LDL	0.931 (0.716-1.209)	0.592	—	—
Uric acid	1.000 (0.997-1.003)	0.979	—	—
Serum tHcy	1.017 (0.992-1.043)	0.186	—	—
Medication				
Antiplatelet Statin Insulin Metformin DPP-4 inhibitors GLP-1 receptor agonist	0.737 (0.457-1.188) 0.853 (0.545-1.336) 1.389 (0.873-2.212) 0.614 (0.361-1.045) 1.167 (0.698-1.952) 1.321 (0.738-2.364)	0.210 0.488 0.166 0.072 0.555 0.348	— — — 0.666 (0.373-1.187) — —	— — — 0.168 — —
TyG index	1.582 (1.123-2.227)	0.009	1.472 (1.003-2.160)	0.048

SBP, systolic blood pressure; DBP, diastolic blood pressure; T2DM, type 2 diabetes mellitus; TC, total cholesterol; HDL, high density lipoprotein; LDL, low density lipoprotein; tHcy, total homocysteine; TyG, triglyceride glucose; CSVD, cerebral small vessel disease.

^1^Adjusted with P < 0.10 in the univariable analysis (age, sex, hypertension, SBP, history of stroke, HDL, metformin usage and TyG index).

There was a significant trend among tertiles of TyG index levels and risk of severe CSVD burden. The OR of severe CSVD burden of the highest tertile of TyG index levels (≥9.22; median 9.58) compared with the lowest (≤8.71; median 8.39) was 2.341 (95% CI: 1.294 to 4.328; *P* for trend = 0.005) after adjusting for age, sex and education. In addition, the OR was attenuated after further adjustment for hypertension, history of stroke, SBP, HbA1c, HDL-C, insulin or metformin usage and serum tHcy, in which the highest tertile of TyG index levels compared with the lowest had an OR of 1.933 (95% CI: 1.010 to 3.698; *P* for trend = 0.047) (**Table 4**).

**Table 4:** ORs (and 95% CIs) of cognitive impairment and severe CSVD burden score according to tertiles of TyG index levels.

	TyG index levels, median (range)			
	Quartile 18.39 (≤8.71)	Quartile 28.96 (8.72-9.21)	Quartile 39.58 (≥9.22)	*P* value for trend ^1^
Cognitive impairment				
Model 1 Model 2 Model 3	1.00 (reference) 1.00 (reference) 1.00 (reference)	1.771 (1.007-3.114) 1.809 (1.002-3.264) 1.748 (0.926-3.299)	4.059 (2.269-7.260) 4.709 (2.515-8.817) 3.298 (1.685-6.452)	< 0.001 < 0.001 < 0.001
Severe CSVD burden score				
Model 1 Model 2 Model 3	1.00 (reference) 1.00 (reference) 1.00 (reference)	1.040 (0.600-1.803) 1.066 (0.603-1.885) 1.082 (0.589-1.986)	1.998 (1.145-3.485) 2.341 (1.294-4.328) 1.933 (1.010-3.698)	0.014 0.005 0.047

^1^Tests for trend were conducted by treating the tertiles as a continuous variable and assigning the median for each quintile.

Model 1: Unadjusted; Model 2: Adjusted for age, sex and education. Model 3: Adjusted for age, sex, education, hypertension, history of stroke, SBP, HbA1c, HDL, insulin or metformin usage and serum tHcy.

### Mediation by severe CSVD burden

**Figure 1:**
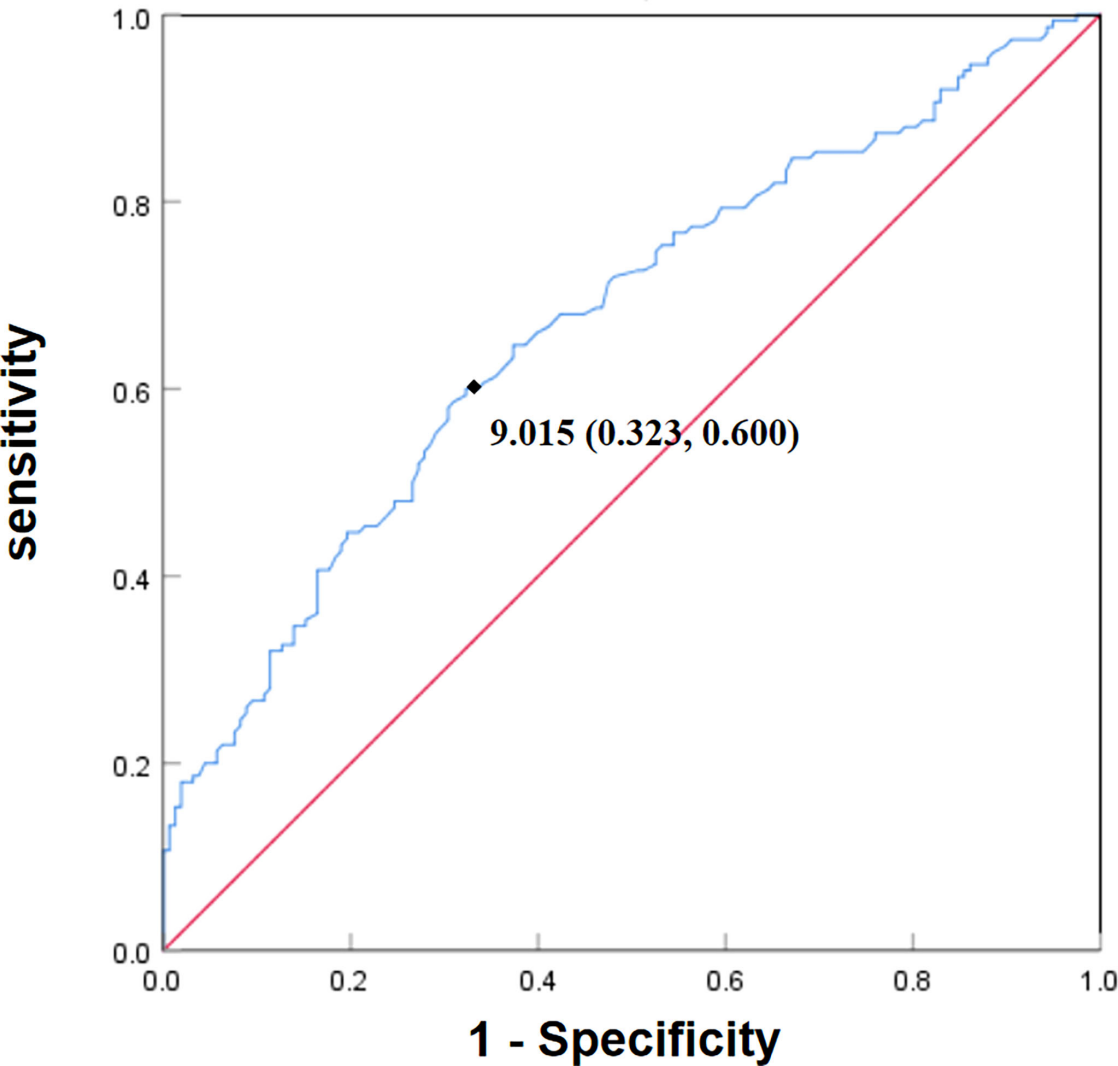
Receiver operating characteristic (ROC) curve of TyG index levels for cognitive impairment. The optimal cut-off point of TyG index levels of patients with cognitive impairment was 9.015. The specificity was 0.677 (1-0.323) and sensitivity was 0.600. The area under the curve (AUC) was 0.671.

The optimal cut-off point of TyG index levels of participants with cognitive impairment was 9.015 and the area under the curve (AUC) was 0.671 (**Figure 1**). To further investigate the association between TyG index and cognitive function, participants were dichotomized into a higher TyG index levels group and a lower TyG index levels group using the optimal cut-off point.

**Figure 2:**
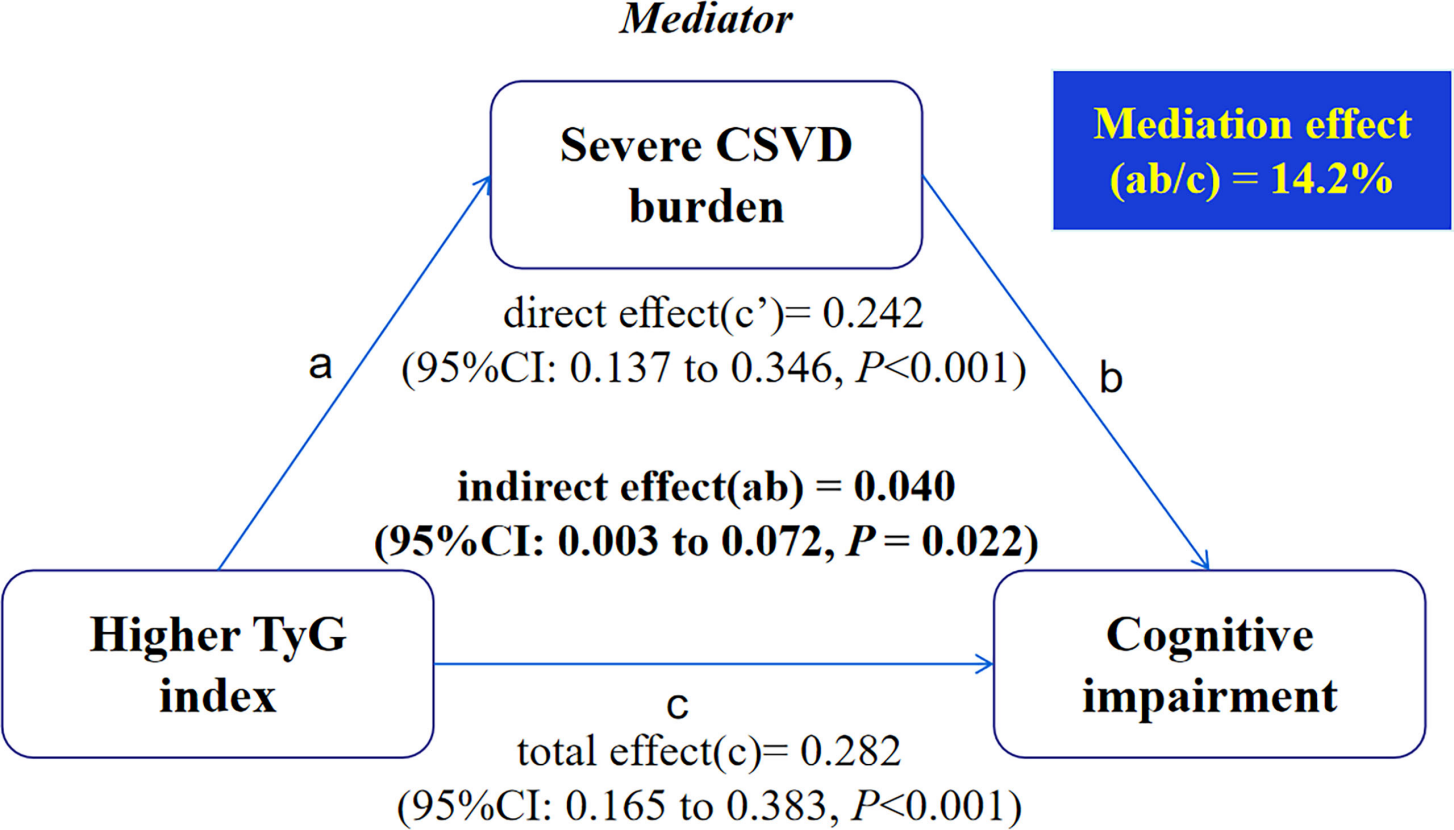
Mediation analysis is shown for the presence of severe CSVD burden as a mediator in the relation between higher TyG index and cognitive impairment.

The result of the mediation analysis is presented in **Figure 2**. The direct effect (c’) and total effect (c) were significant present between higher TyG index levels and cognitive impairment. When severe CSVD burden was added to the model, the indirect (i.e., mediating) effect (ab) of severe CSVD burden was significant for the association between higher TyG index levels and cognitive impairment and 14.2% of the total effect was due to mediation by the presence of severe CSVD burden after adjusting for potential confounders (ab = 0.040; 95%CI: 0.003 to 0.072, *P* = 0.022).

## Discussion

In this retrospective study, we explored the association of TyG index with cognitive function and CSVD burden in elderly patients with T2DM. We found that elevated TyG index was associated with an increased risk of severe CSVD burden or cognitive impairment in elderly patients with T2DM. These positive associations tended to be stronger in those with higher TyG index levels. In addition, we found the presence of severe CSVD burden mediated the effect of higher TyG index levels on cognitive impairment, which may support the hypothesis that increased TyG index may aggravate CSVD, which, in turn, increase the risk of cognitive impairment.

Several studies have suggested that increased TyG index was associated with the development of different imaging markers of CSVD (17, 25, 26). It has been reported that TyG index was associated with the volume of WMH and silent brain infarcts in a healthy population (25). In addition, a recent retrospective study found that increased TyG index was an independent risk factor for moderate to severe EPVS (17). However, as far as we know, no study had explored the association between TyG index and CSVD burden score, which may be more representative of CSVD (9). We observed the levels of TyG index were positively associated with the severe CSVD burden and stronger associations in the highest tertile of TyG index levels compared with the lowest, which suggested that increased TyG index may accelerate the progression of CSVD. Although the exact mechanism between TyG index and CSVD remains elusive, several possible explanations have been proposed. Increasing evidence suggests that brain insulin resistance can lead to endothelial dysfunction, BBB dysfunction, oxidative stress, chronic inflammation and arteriolosclerosis due to failure of cells to respond to insulin (10, 27–29). Interestingly, these mechanisms have proven to be important factors for the pathogenesis and development of CSVD (12–14). Moreover, it is recognized that TyG index is a reliable and alternative marker for insulin resistance (30, 31). Therefore, these above mechanisms may be contributed to the relationship of TyG index and CSVD.

T2DM-related cognitive impairment is an important complication of T2DM and has attracted increasing attention in recent years. However, the underlying mechanism remains unclear and is a challenge for clinicians. An increasing body of preclinical and clinical evidence suggests that insulin resistance is emerging as an important mechanism of cognitive impairment in patients with T2DM (10, 11, 32–34). Brain insulin resistance is defined as the failure of cells to respond to insulin in the brain, resulting in dysfunction of synaptic, metabolic and immune response (10, 33). Subsequently, iron deposit, calcium dyshomeostasis, oxidative stress, inflammation, endothelial and BBB dysfunction occur in the brain, which eventually contribute to cognitive impairment (10, 11, 28). Two studies indicated that manipulating brain insulin directly with intranasal insulin administration restores neural connectivity and improves cognitive function in patients with T2DM (35, 36). In our study, we found that raised TyG index, an alternative marker of insulin resistance, was associated with an increased risk of cognitive impairment in aged patients with T2DM, which provides evidence for the role of insulin resistance in cognitive impairment.

Another important underlying mechanism of T2DM-related cognitive impairment might be cerebral microvascular dysfunction (7, 37), which is widespread in patients with T2DM (38). Pathological changes of cerebral microvasculature in T2DM include basement membrane thickening, increased angiogenesis, increased BBB permeability and altered microvascular blood flow regulation, which lead to perfusion defects, hypoxia, oxidative stress, inflammation and increased angiogenesis (7, 37). These changes could contribute to cognitive impairment in patients with T2DM. It is commonly recognized that imaging markers of CSVD reflect cerebral microvascular dysfunction (12). Additionally, CSVD itself is a prominent cause of cognitive impairment in the elderly (12). In this study, we also found that severe CSVD burden was associated with an increased risk of cognitive impairment in elderly patients with T2DM, which provides evidence for the role of cerebral microvascular dysfunction in cognitive impairment of T2DM patients.

Strangely, few studies have taken CSVD into account when exploring the relationship between insulin resistance and cognitive impairment in elderly patients with T2DM, although they share similar pathogenic mechanisms in cognitive impairment. In our study, we found that raised TyG index or severe CSVD burden was associated with an increased risk of cognitive impairment in elderly patients with T2DM after controlling for potential confounders. Furthermore, we found the presence of severe CSVD burden mediated the effect of higher TyG index levels on cognitive impairment, which may support the causal pathway in which increased TyG index aggravated the development of CSVD, which, in turn, increased the risk of cognitive impairment.

The main strength of our study is that we took CSVD into account when analyzing the relationship between insulin resistance and cognitive impairment in elderly patients with T2DM. Another advantage is that we used TyG index, a convenient and accessible indicator for insulin resistance, to explore the association between insulin resistance and cognitive function or CSVD burden in elderly patients with T2DM.

There are still some limitations. First, the retrospective design of this study limits the ability to validate the causality between TyG index and cognitive function or CSVD burden. Second, this was a relatively small sample study, which was conducted in a single center. Third, other potential confounders, such as APOE genotype were not considered. They may bias our analysis in the study. Finally, patients with mild cognitive impairment and dementia were not distinguished in the current study. Future follow-up studies are required to address these issues.

## Conclusions

In conclusion, our study provides evidence that TyG index levels are positively associated with the risk of cognitive impairment or CSVD burden in elderly patients with T2DM. More importantly, the presence of severe CSVD burden shows a significant moderating effect on the relationship between higher TyG index levels and cognitive impairment. These results suggest that a portion of the effect of increased TyG index on cognitive impairment can be due to the aggravation of CSVD burden.

## Data availability statement

The raw data supporting the conclusions of this article will be made available by the authors, without undue reservation.

## Ethics statement

The studies involving human participants were reviewed and approved by Ethical Committee of Hebei General Hospital. Written informed consent for participation was not required for this study in accordance with the national legislation and the institutional requirements.

## Author contributions

PL designed the study and revised the manuscript. ZT and JF analyzed the data and wrote the manuscript. YD and HC performed the collection of samples and data. JX, XJ and QQ made contributions to data acquisition and analysis. RL and WC made suggestions for the revision of the manuscript. All authors contributed to the article and approved the submitted version.

## Conflict of interest

The authors declare that the research was conducted in the absence of any commercial or financial relationships that could be construed as a potential conflict of interest.

## Publisher’s note

All claims expressed in this article are solely those of the authors and do not necessarily represent those of their affiliated organizations, or those of the publisher, the editors and the reviewers. Any product that may be evaluated in this article, or claim that may be made by its manufacturer, is not guaranteed or endorsed by the publisher.
